# Experimental study on physiological responses during interval exercise and the effects on thermal perception

**DOI:** 10.1007/s00484-025-02993-6

**Published:** 2025-07-24

**Authors:** Yujie Lin, Hong Jin, Tingkai Yan, Jian Kang

**Affiliations:** 1 College of Landscape Architecture, Northeast Forestry University, Harbin, 150040 China; 2 School of Architecture and Design, Harbin Institute of Technology, Harbin, 150006 China; 3 School of Architecture and Design, China University of Mining and Technology, Xuzhou, 221116 China; 4 Institute for Environmental Design and Engineering, The Baetlett, University College London (UCL), London, WC1H 0NN UK

**Keywords:** Interval exercise, Physiological responses, Dynamic thermal perception, Thermal chamber experiments, Thermal sensation model

## Abstract

The physiological response during interval exercise and its significance in determining thermal perception remain unclear. There is a paucity of quick and convenient thermal perception assessment methods in exercisers, which impedes the advancement of real-time thermal perception evaluation in exercise spaces. In this study, five thermal conditions were set up in a thermal chamber to simulate the thermal environment of a cold region and evaluate the dynamic physiological responses during interval exercise and their effect on temperature perception.The results showed that heart rate, sweat feeling index, and heat storage rate values were in line with thermal sensation dynamics. The calf’s skin temperature had the strongest correlation with thermal perception among the other skin temperatures. While heart rate was tightly linked to metabolic heat production, the calf’s skin temperature was affected by both exercise intensity and ambient temperature, which are directly related to thermal perception during interval exercise, and these two physiological can be tracked in real-time with portable devices. A model for predicting thermal perception was developed based on heart rate and the skin temperature of the calf, and the explanation rate was 89.8%.

**Supplementary Information:**

The online version contains supplementary material available at 10.1007/s00484-025-02993-6.

## Introduction

Interval exercise refers to a exercise method that alternates between high-intensity activities, low-intensity activities and recovery periods. Compared to continuous exercise, interval exercise is more common in daily life and work, and may enhance physical fitness, endurance, and metabolic rate effectively (Zhen et al. 2025). However, the significant fluctuations in thermal load during interval exercise, along with the alternating intensity and duration, make the dynamic changes in temperature regulation, therefore influence physiological responses and thermal perception (Jones and Vanhatalo 2017).

The main effects of physiological regulation functions via metabolic heat production in response to changes in temperature, with relevant factors including skin temperature (Tskin), heart rate (HR), heart rate variation(HRV), heat storage rate (HSR), and sweat feeling index (SFI) (Li et al. 2022; Wang et al. 2022; Lin et al. 2023). Psychological adaptation mainly refers to differences in subjective perception at the psychological level, including thermal perception votes, thermal sensation votes (TSV), and thermal acceptance votes (Lin et al. 2022). Physiological indicators reflect the physical condition and intensity at which individuals are exercising. Thus, they are potential indicators for assessing the thermal perception of individuals during interval exercise.

As reported in the literature, Tskin and thermal perception are correlated (Vanos et al. 2010). Takada et al. proposed a new model for predicting TSV in the unsteady state based on Tskin, and developed multiple regression equations for transient thermal sensation versus mTskin and its time difference based on experimental data from participants in a sedentary state (Takada et al. 2013).Lai et al. found the average Tskin and its change rate were indicators of the dynamic influences of the environment on the thermal perception of the body (Lai et al. 2017). Li et al. identified the Tskin of the wrist as a potential predictor of TSV in subjects with different levels of activity intensity (Li et al. 2018). Zhang et al. found that the temperature of the ear canal in individuals during walking decreased at the beginning of the exercise period and then increased after the exercise period (Zhang et al. 2020b). Li et al. found that during the exercise, the mTskin value decreased rapidly in the first 5–8 min and gradually increased in the remaining time; at the end of the exercise, the mTskin value continued to show an increasing trend in the first 8–10 min before gradually decreasing in the remaining time (Li et al. 2022). Xu et al. Found that the Tskin of the chest fluctuated less than the Tskin of the calves and arms in sedentary participants (Xu et al. 2022).

Sweating is a very effective way of heat dissipation in human heat regulation. Wang et al. found that SFI and thermal perception were correlated when the human body exercise in a hot environment (Wang and Hu 2014). Wang et al. studied the thermal sensation of moderately active participants via experiments, and the results showed that the thermal regulation process of sweat has some influence on TSV and mTskin, leading to a decrease in neutral Tskin during moderate activity (Wang and Hu 2016). Li et al. recorded SFI rather than skin moisture or sweating rate to determine sweating of human, and determined a positive linear relationship between TSV and SFI (Li et al. 2022).

Heart rate reflects the activity intensity and physiological state of the human body, and some scholars have studied the relationship between HR and thermal perception. In addition, among the physiological indicators related to HR, HRV has attracted more attention, which is a physiological indicator reflecting the cycle changes of human heart rate. Yao et al. found that, HRV was higher when the participants stayed in the cold or hot environment, and was the lowest when they stayed in the pleasant thermal environment(Yao et al. 2009). A quantitative relationship between heart rate and TSV values in the temperature range of 30–39℃ was established by Liu et al. (Liu et al. 2021).

Although some studies have investigated the relationship between physiological responses and thermal perception, the majority have focused on individuals under conditions of sedentary or stable exercise in low- to moderate-intensity, produced prediction models of thermal perception with a narrow range of applications (Sim et al. 2016; Wang and Hu 2016; Ghahramani et al. 2016). This study set up five groups of temperature conditions to simulate the climate of a cold region city, with a temperature range of −5 ~ 35℃, aimed to address the following three research questions using thermal environment chamber experiments: (1) What are the dynamic characteristics in physiological responses during interval exercise in different thermal environments? (2) What are the effects of physiological responses on thermal perception during interval exercise? (3) How to achieve real-time evaluation of interval exercise through simple indicators? The results of the study provide a theoretical basis and empirical support for the monitoring of physiological indices and the prediction model of thermal perception in individuals during moderate- and high-intensity interval exercise.

## Methodology

### Experimental design

**Fig. 1:**
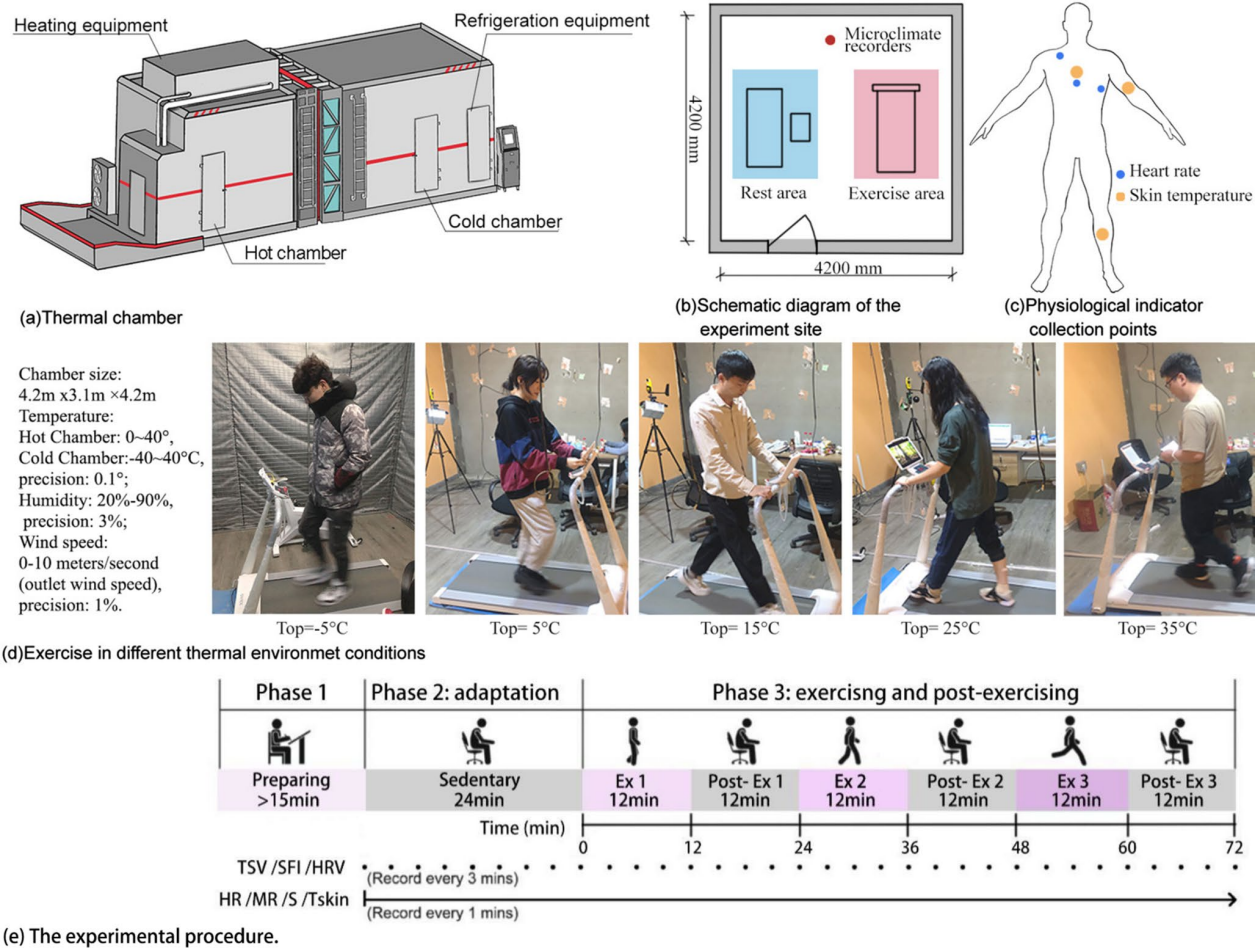
Experimental design and procedure

This study was carried out in Harbin, Heilongjiang Province, China, which belongs to the hot summer continental (Dwa) of Koppen climate classification, with a temperature difference of up to 40 °C throughout the year. The thermal chamber in the cold region experiment center of Harbin Institute of Technology was used to simulate the thermal environment. This system provided a controllable thermal environment for research, effectively shortening the research cycle and improving the accuracy of research (Fig. 1). This study established five groups of thermal conditions (operative temperature ranged from − 5 to 35℃) to assess the effects of thermal environment, and the parameters in chambers were automatically controlled during the experimental process.

All the participants of this study were college students studying in Harbin and had lived there for more than one year. Participants were enrolled in all experimental sessions, and they were asked to refrain from smoking, drinking alcohol or coffee, and performing strenuous exercise 24 h before the experiments. Respondents were asked to wear clothing suitable for the set temperature for the experiment, and clothing was also provided for changing. The mean values of the thermal resistance of the subjects’ clothing at −5, 5, 15, 25, and 35 °C were 1.5, 1.3, 1.0, 0.7, and 0.5 clo, respectively, which complies with the clothing thermal resistance of different thermal environments in Harbin (ISO 9920 2007; Jin et al. 2019). The experiment of this study was conducted in accordance with the Declaration of Helsinki and received human ethical approval from the institution.

Taking into account the statistical power level and experimental conditions, a total of 120 healthy college students voluntarily participated in the experiment, of which 48% were female and 52% were male (Table 1). A total of 2,961 sets of valid data were obtained, with each dataset containing subjective thermal perception votes, five points of skin temperatures, SFI, HR, HRV, and HSR (Lin et al. 2023).

**Table 1:** Anthropometric data of participants in this study

Gender	Age(year)	Height(m)	Weight(kg)	BMI (kg/m^2^)	BMR (kcal/day)	ADu
Males	24.7 ± 1.3	1.76 ± 0.04	72.1 ± 9.4	22.4 ± 1.9	1700.6 ± 137.2	1.87 ± 0.12
Female	26.0 ± 3.2	1.66 ± 0.05	56.6 ± 9.1	20.5 ± 1.8	1329.1 ± 139.6	1.61 ± 0.13
Total	25.3	1.71	65.0	22.1	1530.3	1.75

### Experimental procedure

For the experimental procedure, the movement and rest areas were set up in the thermal chamber. A treadmill (XQiao Q1SP; Shanghai Wenjia Inc., PRC) was used in the exercise area, with a speed range of 0 ~ 12 km/h and an adjustment accuracy of 0.1 km/h. Seats and a desk were provided in the rest area. The participants walked at a set speed on a treadmill during the exercise, and rested in the seats during post-exercise. Throughout the entire experiment, an assistant was available to help the participants adjust the speed of the treadmill, as well as to ensure their safety.

The experiment was divided into three phases, and the duration of the entire experiment was approximately 111 min, as shown in Fig. 1(e). Subjects were required to arrive at least 15 min in advance, and the assistant took their basic information and fitted them with skin temperature and heart rate sensors.The exercise and rest time was set to be 12 min (Ji et al. 2018; Zhang et al. 2020b; Wang et al. 2024). During the thermal adaptation phase, the participants were required to sit in the thermal chamber for 24 min in order to acclimate to the thermal environment, allowing their physiological indices to reach sedentary levels. For interval exercise, three sets of walking exercises (including exercise and rest) were performed in sequence, and the intensity of exercise gradually increases: Ex 1 (3 km/h), Post- Ex 1 (rest), Ex 2 (4.5 km/h), Post- Ex 2 (rest), Ex 3 (6 km/h) and Post- Ex 3 (rest). Subjective questionnaires and HRV were recorded every three minutes from the beginning of phase 2 until the end of the experiment; all other physiological indices were recorded every minute.

### Measurements

The instruments was set up in the thermal chamber at 1.7 m above the ground to monitor and record the physical environment data throughout the experiment (Mayer and Höppe 1987), and the specific parameters and insturments were listed in Table 2, and the thermal parameters measured during experiments were listed in Supplementary Table S1.

**Table 2:** Environmental parameters in the controllable thermal chamber

Type	Parameter	Range	Accuracy	Sampling rate
BES-02	Air temperature, relative humidity	–30 to 50℃, 0 to 99.0%	≤ 0.5 ℃, ≤ 3.0%	1 min
Kestrel 5500	Wind speed	0.1 to 40.0 m/s	0.1 m/s	1 min
iButton DS1922L	Skin temperature	–40 to 85℃	± 0.5℃	1 min
Careshine CCS–103	Heart rate/Heart rate variation	30 to 200bmp	± 5bmp or ± 5%	1 min/3min

The questionnaire was comprised of questions aimed at capturing the basic information of the participants (sex, age, height, weight, and clothing), as well as their thermal sensation and sweat feeling index. TSV was evaluated on a seven-point scale (−3: cold, −2: cool, −1: slightly cool, 0: neutral, + 1: slightly warm, + 2: warm, and + 3: hot) (ISO 7730 2005; ASHRAE 2017; Mihara et al. 2019). SFI was categorized as 0, 1, 2, and 3, which indicated that the participants had no feeling, slight feeling, obvious feeling, and strong feeling of sweat, respectively (Li et al. 2022).

The physiological response index data were collected using a portable device, including the local Tskin, HR, and HRV. Previous studies shown that HRV contains information about the autonomic nervous system (Maria et al. 2017). The key parameter LF/HF, which represents the ratio of sympathetic and vagal activity, was used to study human thermal perception (Zhu et al. 2018). The Careshine Electronic heart rate monitor (CCS-103; Careshine Electronic Technology Ltd.) was used to record the HR and HRV of the participants during interval exercise (Xiong et al. 2019), which was stored in the subjects’ pocket and linked to the electrode pads via skin-friendly wires. The electrode pads were placed on three points of human body, as shown in Fig. 1. Tskin was recorded using iButtons DS1922L, which were attached by steam permeable surgical tape to ensure close contact with the subject’s skin. The Tskin were divided into three groups: the core group (including forehead and chest), the intermediate group (including upper legs and calves), and the distal group (including lower arm, back of the hand, and instep), based on their positions in the human thermoregulation system and thermal sensation model (Zhang et al. 2010a, b). In this study, five local skin temperatures were collected, including forehead, chest, back, upper arm, and calf, as shown by the yellow dots in Fig. 1, and the mean skin temperature (mTskin) was calculated for comparative analysis (Yao et al. 2007).

### Data processing

The three-point model was used to calculate the mTskin (Alan 1934; Ji et al. 2017; Lam et al. 2021). The HSR changes in real time with exercise. In this study, the HSR was used to describe changes in the heat balance of the human body. Then, the correlation between the HSR and the psychological response was analyzed during the exercise process. The HSR was calculated with reference to previous studies (Nishi 1981; Dear et al. 1997; Fabbri 2015; Yang et al. 2016; Sakoi et al. 2018; Zhang et al. 2020a).

The metabolic rate is usually calculated based on ventilation (Ji et al. 2018) or the heart rate (Zhang et al. 2020b). For the former, most current instruments require individuals to remain at rest and wear unperceptionable masks. This makes it unsuitable for the measurement of motion activities and for field studies (Kristen et al. 2015). By contrast, the latter is more suitable for participants who are exercising. Therefore, in this study the heart rate method was used to calculate the metabolic rate, according to ISO 8996 (ISO 8996 2021).

In this study, the mean values of the individual monitoring data and the corresponding questionnaire results were averaged for correlation analysis of each physiological index using thermal sensation and regression fitting analysis. Spearman was chosen for correlation analysis; the correlation was considered significant at the 0.05 level. Excel was used for data counting, categorization, and preprocessing. Statistical analyses were performed in SPSS version 22.0 (IBM).

## Results

### Thermal responses during interval exercise

1. Thermal sensation votes

**Fig. 2:**
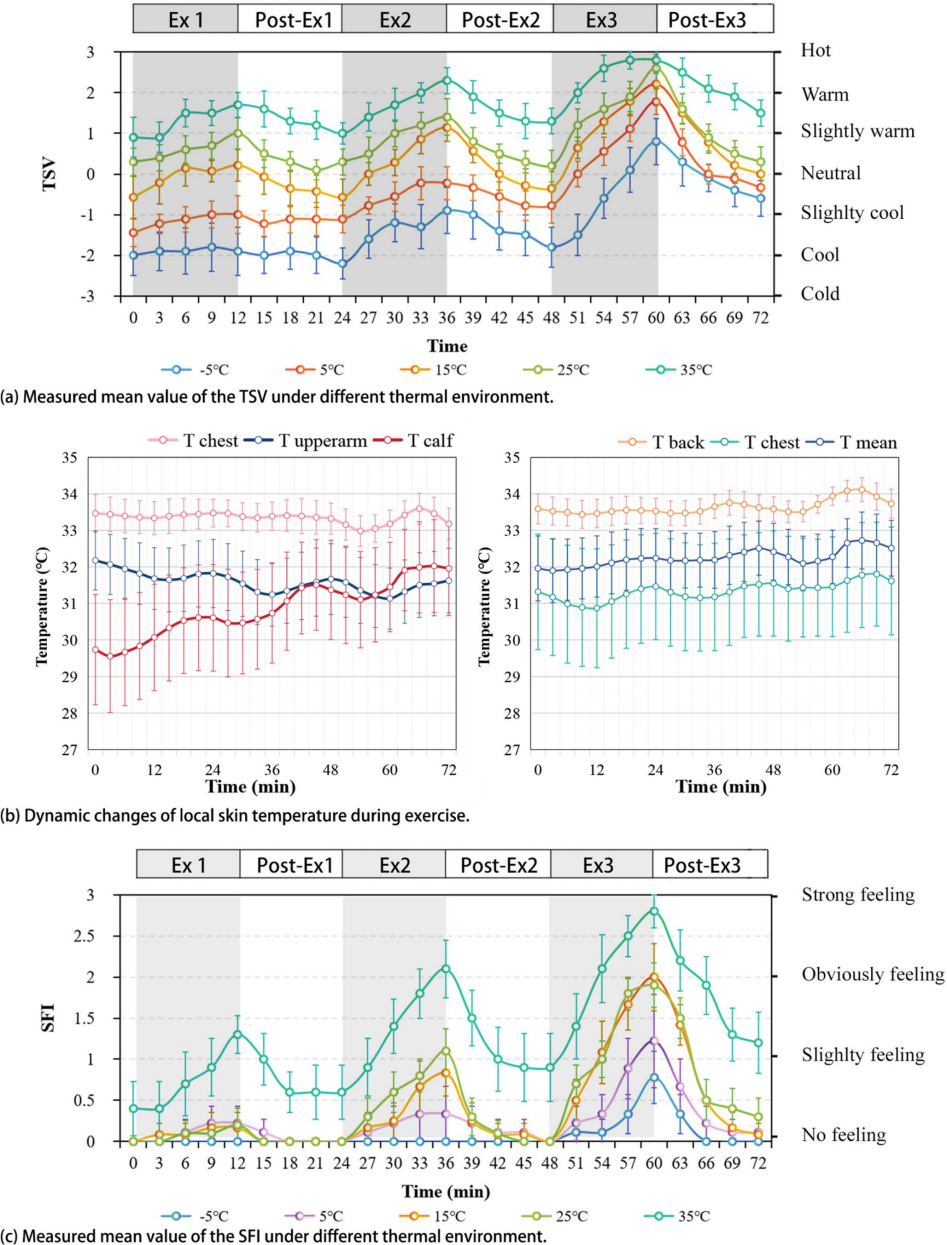
Measured mean value of the TSV, Tskin and SFI under different thermal environment

Figure 2(a) depicts the dynamic changes in TSV among participants in various thermal environments during the experiment. In phase 2, subjective thermal sensation gradually adapted to the environment. The mean TSV for participants exposed to temperatures of − 5℃ to 35℃ was − 1.6, − 1.2, − 0.4, 0.4, and 1.3, respectively.

In phase 3, during interval exercise, the TSV gradually increased after the start of the exercise, and the TSV of Ex 1, 2, 3 increased by 0.5, 1.3, and 2.2, respectively. TSV gradually decreased after exercise cessation and recovered to near baseline levels at the end of Post-Ex1 and 2, and was slightly higher than baseline at the end of Post-Ex3, and the average increment in TSV was 0.4.

It can be seen that ambient temperature and the level of exercise intensity have an intuitive effect on thermal perception.Taking the moment at the end of the exercise as an example, a 5 °C increase in the temperature environment increases the TSV by about 0.6 (S.D. = 0.258), and a 1.5 km/h increase in walking speed increases the TSV by about 0.9 (S.D. = 0.307).

(2) Skin temperature

With regards to changes in the local skin temperature, a plot of the fluctuations in the skin temperature at each site during the experiment was provided in Fig. 2(b). The average value of the local skin temperature in different temperature environments was used to determine the overall change characteristics of the local skin temperature over the course of the experiment.

First of all, the local skin temperature was characterized by a different degree of fluctuation dynamics during interval exercise, which was manifested as follows: it first decreased and then increased during Ex 1, 2, and 3; then, during the post-exercise phase, continued to increase for about 6 min, followed by a period of recovery. Skin temperature and TSV changed in the opposite direction once the activity state started to change. Second, the degree of variation in local skin temperature varied widely. The average standard deviation of the skin temperature on the chest, back, upper arm, calf, and forehead over the course of the experiment was 0.79, 0.75, 2.21, 3.09, and 3.18, respectively, indicating that in the interval exercise, the skin temperature of the calf and forehead fluctuated the most drastically, while those of the chest and back were the most stable. Finally, the overall trends in Tskin were not the same across the different parts of the body. Compared to the Tskin at the beginning of the experiment, the Tskin of the calf showed a significant increase in temperature at the end of the experiment (+ 2.46 °C), while the temperature changes in the other parts of the body were smaller. This indicates that the Tskin of calf exhibits significant numerical differences due to variations in exercise intensity, while no significant trend was observed in other parts of the body (including mTskin).

(3) Sweat feeling index

Figure 2(c) shows the dynamic changes in SFI during the experiment in the participants in the different environments: SFI increased during exercise and gradually decreased after the beginning of the post-exercise phase. This indicates that dynamic changes in SFI are highly synchronized with TSV. Both temperature and exercise affected SFI, which increased with exercise intensity or ambient temperature. It is worth noting that SFI was significantly higher at 35 °C, and the SFI value before the start of Ex 1 (i.e., at the end of the thermal adaptation) was 0.4, indicating that the subjects already had a sense of sweating. This is because when the ambient temperature is higher than the skin temperature, body regulation leads to cooling by sweating (Vanos et al. 2010).

(4) Heart rate and heart rate variation

**Fig. 3:**
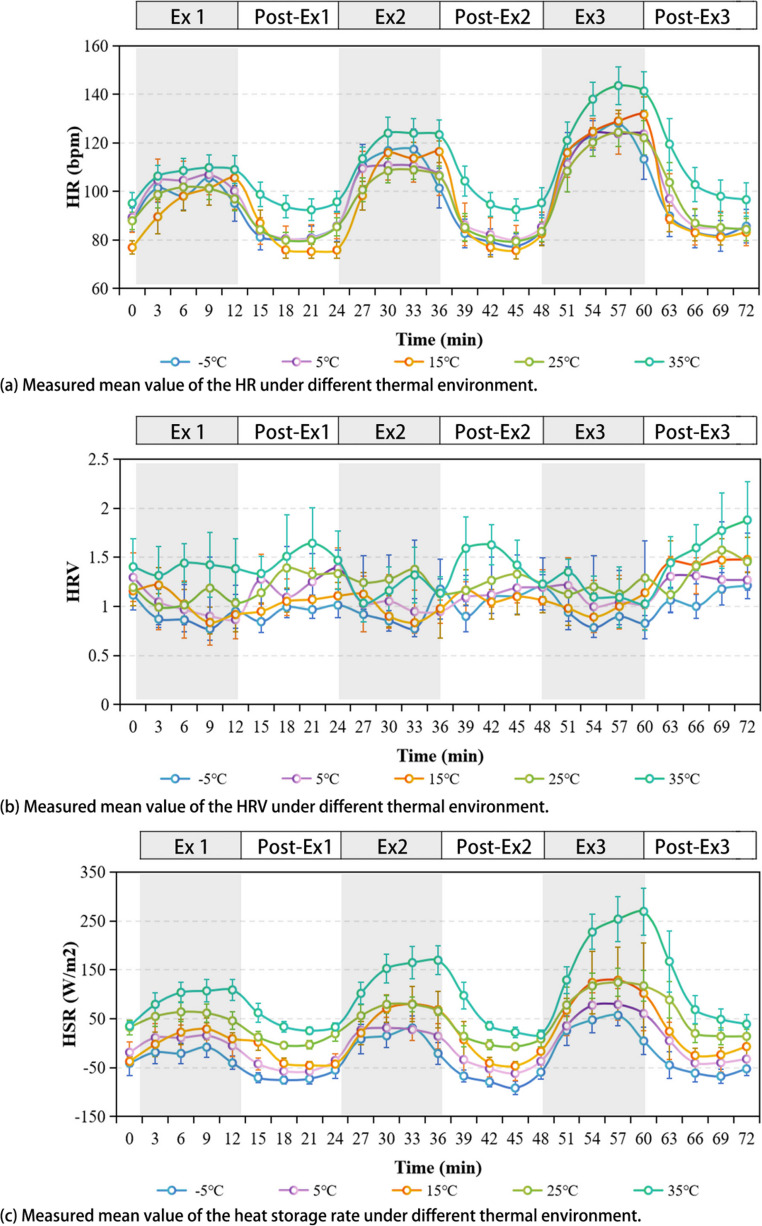
Measured mean value of the HR, HRV and HSR under different thermal environment

The temporal fluctuations in HR were illustrated in Fig. 3(a). Observations indicated an initial rapid ascent in HR at the beginning of the exercise, followed by a progressive stabilization. And then a rapid decline in HR was noted upon the cessation of exercise, culminating in a gradual return to baseline levels. Notably the dynamic characteristics of HR and TSV exhibits a heightened degree of synchrony in all temperature sets. The thermal environment’s impact on HR was not significance. In contrast, the intensity of the exercise was identified as a potent determinant of HR alterations.

The changes in HRV over the course of the experiment for each temperature environment were depicted in Fig. 3(b). The dynamic synchronization between HRV and TSV was weak. The physiological indices reached a relatively steady state at 9 to 12 min of Ex 1, 2, and 3 and Post-Ex 1, 2, and 3. The average of HRV at these two time points was taken to analyze the differences between exercise and rest, as shown in Supplementary Fig. S1. Temperature, exercise intensity, and activity status all had an effect on HRV. Post-Ex 1, 2, and 3 had a higher HRV than Ex 1, 2, and 3 (the average difference was 0.3, S.D.=0.12); for Ex 1, 2, and 3, a higher temperature and exercise intensity resulted in a higher HRV. Ex 1 and Ex 2 had closer HRV, while the HRV for Ex 3 was significantly higher than Ex 1 and Ex 2.

(5) Heat storage rate

Dynamic changes in the HSR of the participants under different exercise intensities and thermal environments were analyzed, as shown in Fig. 3(c). The HSR varied significantly with the duration of exercise, increasing significantly during the exercise phase, decreasing rapidly after exercise stopped, and gradually returning to the level before exercise began. The results suggest that HSR was well synchronized with the dynamic characteristics of TSV. In the environment ranging from − 5 °C to 15 °C, the HSR at the onset of exercise was negative, attributed to the low ambient temperature. The heat lost by the body through evaporation, radiation, and convection exceeded the metabolic heat production. At 25 °C, the HSR was nearly zero at the start of exercise, indicating that the human body was approximately in thermal balance in a sedentary state. In a 35 °C environment, the HSR was positive at the beginning of exercise because the ambient temperature was higher, and the body’s metabolic heat production surpassed the heat loss due to evaporation, radiation, and convection. The extent of HSR change during EX 1, 2, and 3 correlated with the exercise intensity. The greater the intensity, the more significant increment in HSR. At the end of EX 1, 2, and 3, the mean increases in HSR were 51.6 W/m², 87.5 W/m², and 137.5 W/m², respectively.

### Effects of physiological responses on thermal perception

**Fig. 4:**
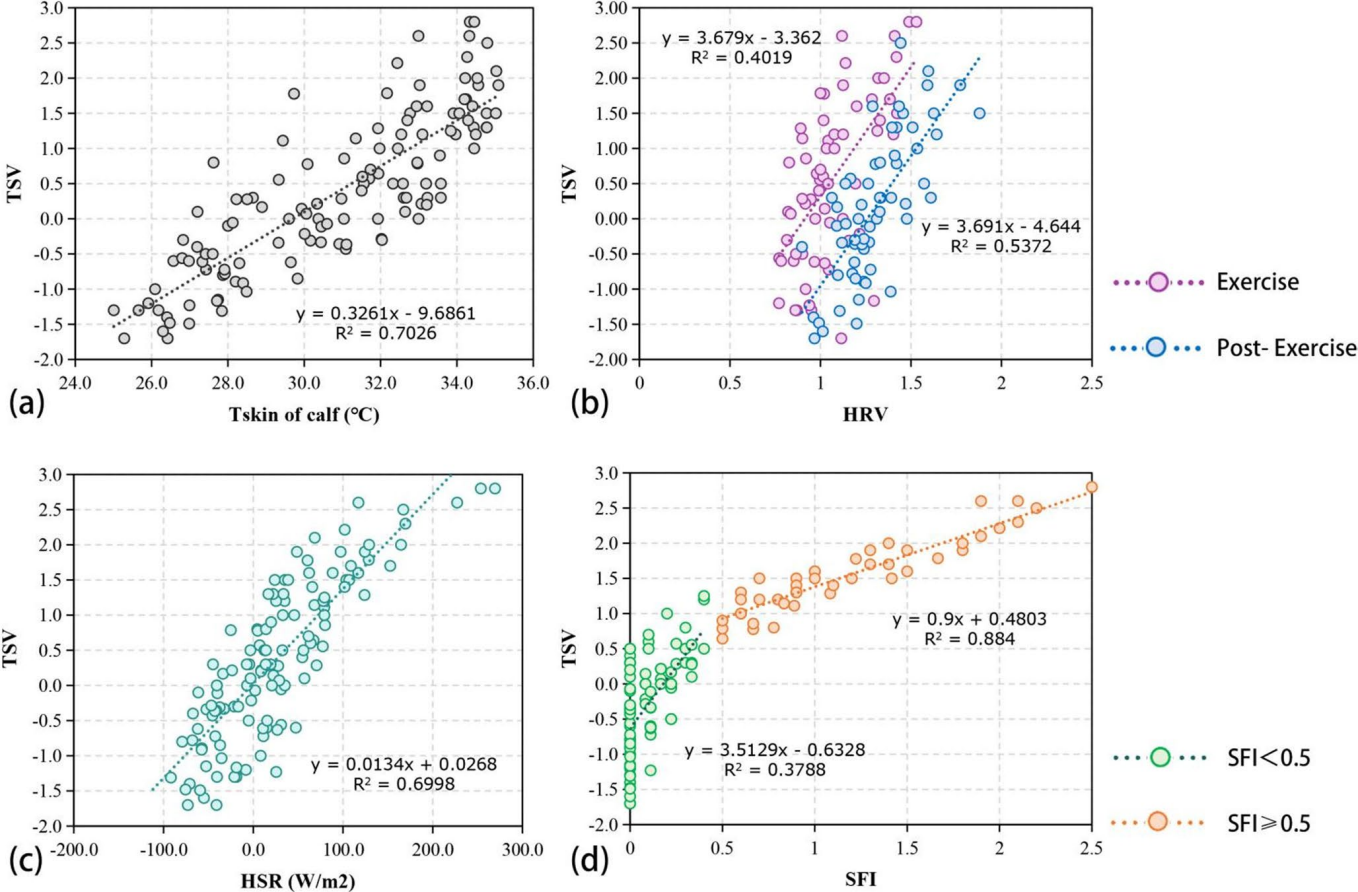
Plot of the linear fit of the (**a**) Tskin of calf, (**b**) HRV, (**c**) HSR, (**d**) SFI and TSV

To determine the effects of physiological responses on thermal perception, the correlation between physiological response and TSV was analyzed (Supplementary Table S2). TSV was found to be significantly and positively correlated with the local skin temperature of each different part of the body. The correlation coefficient between calf and TSV was the highest, and both exercise state and intensity were found to have effects on this part. This suggests that Tskin of calf may be a good potential indicator of thermal perception. A linear regression of the calf skin temperature and TSV was depicted in Fig. 4(a): a uni-variate linear equation with an R^2^ of 0.7026 was obtained, indicating that the proportion of TSV of the subjects during exercise that can be explained by calf skin temperature is 70.26%.

TSV was significantly correlated with HR. Although the correlation between them was low, the dynamic synchronism between them was high. Regarding HRV, a significant positive correlation was observed between HRV and TSV, while the correlation coefficient was low. By contrast, when the post-exercise and exercise states were regressed separately in a linear fashion, as shown in Fig. 4(b), the R^2^ improved; however, the equation explained no more than 60% of TSV. Although HRV reflects the intrinsic regulation of the body’s autonomic nervous system, its poor dynamic synchronism with TSV and low regression R^2^ do not provide a better prediction of thermal perception in individuals during exercise.

TSV followed the same trend as the HSR. In six sets of thermal conditions. A correlation analysis of TSV and HSR indicated that they were positively correlated. The HSR was fit linearly to TSV, as depicted in Fig. 4(c). A coefficient of determination R^2^ of 0.6998 indicates that the proportion of variation in the participants’ thermal sensation that can be accounted for by the HSR is 69.98%.

The trends of SFI and TSV were very similar during the experiment. A significant positive correlation was observed between SFI and TSV, with a correlation coefficient of 0.883. A linear fit was performed for them as shown in Fig. 4(d). When SFI was 0–0.5, the regression equation R^2^ was small, and when SFI was 0.5 and above, the regression equation R^2^ was large; thus, SFI was linearly and positively correlated with TSV. These results suggest that the accuracy of estimating the subjective thermal sensation of the human body using SFI is low when the human sweating sensation is not greater than 0.5; when the human sweating sensation is high than 0.5, the accuracy of estimating the subjective thermal sensation of the human body by SFI is greatly increased.

### Prediction model of thermal perception for exercising individuals

The first step was the selection of the independent variables. As shown in Supplementary Table S2, there were significant correlations between Tskin, HR, HRV, HSR, operative temperature, SFI, and TSV.

Among them, the value of HSR requires complex calculations and there are too many covariates that need to provide data, so it is not suitable to be the independent variable option from the perspective of simplifying the independent variables; The sweat sensation index can hardly predict TSV when the value was less than 0.5 (e.g. low temperatures, low level exercise). The correlation coefficient between Tskin, T_op_ and TSV was relatively high, but the T_op_ had a strong collinearity with Tskin (VIF > 3 and Tolerance < 0.1), and only one of them can be selected as the independent variable. Tskin, HR and HRV can be easily worn and monitored in real time. The correlation between calf skin temperature and TSV was the strongest among the local skin temperature, and there was a significant correlation between heart rate and TSV during exercise, and the dynamic characteristics were more consistent, and there was a covariance between HRV and calf Tskin (the Condition Index of Collinearity Diagnostics was bigger than 10). Therefore, Tskin and calf HR were used as the two independent variables in the TSV evaluation formula, and TSV was used as the dependent variable in the multiple linear regression analysis. The results are shown in Eq. 1.

1 $$TSV=0.297\ast Tskin_{calf}+0.03\ast\;HR-11.786\;(R^2=0.898)$$

**Fig. 5:**
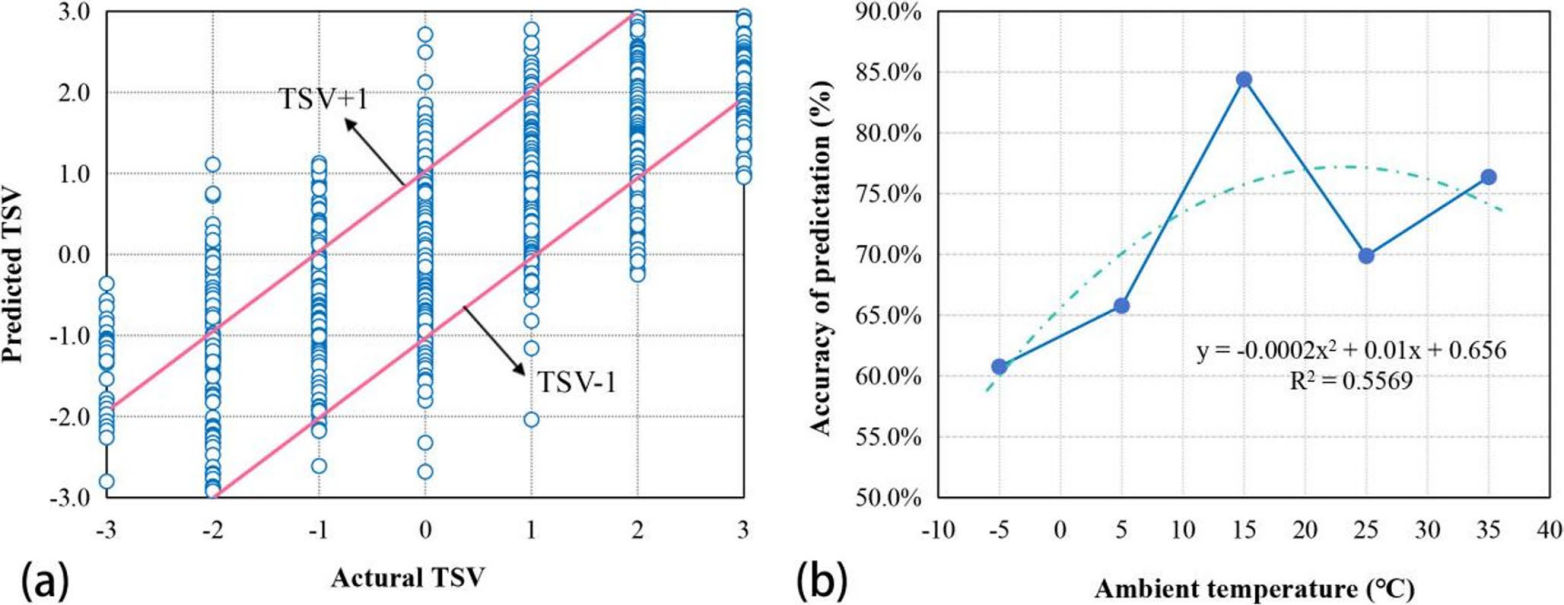
(**a**) Comparison between actual TSV and predicted TSV for individuals; (**b**) Correlation of prediction accuracy with ambient temperature

Figure 5(a) presents a comparison between the actual TSV and the predicted TSV. Two deviation lines were depicted to illustrate the dispersion trend of the predicted TSV. The findings indicated that in 72.3% of the data, the discrepancy between the predicted and actual thermal sensation was less than one unit. Given the intricacies of physiological regulation and psychological adaptation, these predictions are deemed acceptable (Xu et al. 2022). As depicted in Fig. 5(b), there exists a correlation between the accuracy of the equation’s predictions and ambient temperature. The prediction accuracy varies across different ambient temperature, with the lowest accuracy of 60.8% observed at −5℃ and the highest accuracy of 84.4% achieved at 15℃.

## Discussion

In this study, Tskin, HR, HRV, HSR, SFI were selected as physiological indicators, which may potentially affect thermal perception, and the dynamic characteristics in the ambient temperature ranged from − 5 to 35℃ were analyzed. Through correlation analysis, the physiological indicators that can predict TSV were chosen. An evaluation model of TSV based on HR and Tskin of calf was established by multiple linear regression analysis.

Compared with the previous studies, the results of this paper deepened the theoretical and experimental studies of the thermal perception of exercising individuals. However, the exercise intensity, living habits, living area and climate background of the participants have a certain impact on the physiological and psychological response, so the results of this paper are also different from the previous studies.

### Dynamic characteristics of physiological responses in interval exercising

In this study, the skin temperature gradually decreased after the start of exercise, and then continuously increased after the exercise stopped, and gradually recovered. This is more consistent with the findings of Li et al. and Zhang et al.(Li et al. 2022) (Zhang et al. 2020b). The reason that skin temperature presented such dynamic features during movement is that, at the initial of walking exercise, heat production in the human body had not covered the loss of heat from the enhancing convective heat transfer through the skin due to walking, and the core thermogenesis increases and gradually passes to the skin surface. After the movement was stopped, continuous heat transfer, the skin temperature gradually decreases after continuing to increase for some time. In addition, Li et al. studied the average skin temperature. In this study explored the dynamic trend of skin temperature in different areas during movement, and showed that the fluctuation amplitude of local skin temperature during movement was quite different. Among them, the skin temperatures of the calf increased significantly with the increase of the exercise intensity during the experiment, while those of the upper arms did not show a significant upward trend or even decreased a little, which was attributed to the fact that the skin temperatures of the lower half of the body increase due to the heat generation from the muscle activity, while those of the upper half of the body decrease due to the convective heat loss (Takada et al. 2013).

In this study, a higher exercise intensity caused an increase in the SFI, and it took longer to return to the initial SFI levels. For Ex 1, 2, 3 at 35 °C and Ex 3 at 25 °C, recovery did not occur under some conditions. The dynamic changes of SFI during exercise are relatively similar to Wang’s findings(Wang et al. 2024), based on which the present study found that the faster the walking speed, the longer the SFI recovery time, and in higher temperature environments (25, 25 °C), the SFI takes longer to recover. In previous studies, Zhang et al. demonstrated the dynamic correlation between thermal storage rate and TSV(Zhang et al. 2020b).

### Physiological responses and the effects on TSV

This study investigated the evaluation and prediction of thermal perception of exercising individuals. Physiological and thermal perception responses were recorded in a temperature range of − 5 to 35 °C during and after exercise. Heart rate and the skin temperature of the calf were selected as independent variable indices of the exercisers’ thermal sensation evaluation model, and fitting regression produced a model with a high prediction accuracy. An increasing number of researchers are using human physiological response data as a predictor in the current thermal perception prediction studies, particularly when assessing thermal perception in non-steady physiological and thermal environmental states like light to moderate exercise.

The results of this study indicated that skin temperature was significantly correlated with TSV; the calf and forehead skin temperatures had higher correlation coefficients with TSV, close to 0.9, in agreement with previous studies (Bulcao et al. 2000; Yao et al. 2007; Li et al. 2018). In this study, dynamic synchronization between the heart rate and TSV of the participants was high, and a significant correlation was observed between the two. Although the correlation coefficient was small, adding it as an independent variable in the regression fitting of calf skin temperature and TSV led to an increase in the R^2^ of the evaluated equation from 0.7026 to 0.898, which could improve the prediction accuracy. In previous studies, Chen et al. and Choi et al. demonstrated the possibility of using the heart rate as a parameter to estimate the thermal sensation of the human body (Choi et al. 2012; Chen et al. 2023); however, they did not develop a corresponding mathematical model for practical application. By contrast, this paper proposes a prediction model on this basis, with HR used to improve the accuracy of the model prediction.

### Evaluation model of thermal perception

Regarding prediction of TSV, Takada et al. developed a novel model based on Tskin and its change rate using nonlinear regression analysis (R2 = 0.799) to forecast thermal sensation in the non-steady state (Takada et al. 2013). Lai et al. developed a model for predicting the thermal perception using the thermal load and the change rate of the mean skin temperature, and the model’s R^2^ was 0.811 (Lai et al. 2017). Zhang et al. established thermal perception prediction model based on Tskin of pelvis and change rate of Tskin, and the model’s R^2^ was 0.69 (Zhang et al. 2010a). Su et al. correcfied COMFA model for elderly exercising individuals through experimental studies, and the modified COMFA-Senior model could better explained the changes in heat balance and actual thermal sensation during sitting and walking (R^2^ = 0.71 and 0.77), but could not describe that in dancing (Su et al. 2023).

In the existing study, the model validation temperature ranged from − 7 to 43 °C, and the participants performed low to moderate levels of activity (e.g. sedentary, computer work, meal, and walking at 3.0 met). Accordingly, the thermal perception rating equations presented herein are applicable to low, moderate, and high levels of exercise, as well as to a wider range of thermal environments. The explanation rate of the thermal perception evaluation index based on physiological response in the above studies ranged from 69 to 81.1%. The present study improved the explanation rate of the evaluation model, which can more accurately reflect the thermal perception level of the exercising individuals.

### Practical implications

The results of this study deepen the research on thermal perception of interval exercise, uncovering the dynamic physiological and psychological characteristics of interval exercise and their interrelationships. A thermal perception prediction model was established based on skin temperature and heart rate, and these two physiological parameters that can be obtained in real-time through portable devices. This significantly simplifies the thermal perception prediction process for exercising individuals and provides convenience for application design and planning.

This study contribute to the creation of more perceptionable exercise spaces and can be applied to the evaluation and optimization of thermal perception in existing external facilities. For example, in the design of an exercise space, designers can set up skin temperature infrared monitoring devices and heart rate monitoring devices to calculate the real-time thermal sensation levels. Intelligent space thermal environment control systems can also be developed in conjunction with the needs of the site to appropriately adjust space thermal environment parameters, such as activating fans, spraying water mists, and opening or closing sunshades, based on estimates of the thermal perception of individuals using the facilities.

### Limitations and future works

Firstly, this study focused on moderate to high-intensity walking exercises, so the applicability of the TSV evaluation model to other forms of exercise or different intensities remains to be validated. Secondly, due to the impact of the COVID-19 epidemic, access to the thermal chamber in the college campuses was restricted; therefore, the respondents were all university students ranging in age from 23 to 32 years. Consequently, the results of this study are only applicable to the young population. To further deepen the research, the characteristics of thermal response during exercise in middle-aged and older adults need to be investigated, and more exercise types shall be added in the study (e.g., limb movements and court games), and outdoor experiments need to be conducted with physiological and psychological data collection, and the model’s performance in real-world outdoor environments needs to be tested in the future works.

## Conclusions

To study the dynamic characteristics of physiological responses during interval exercise and their effects on thermal perception, college students were recruited to exercise in different intensities and thermal conditions, while monitoring and recording real-time physiological responses and thermal sensation votes during interval exercise. The main findings of this paper are as follows.

1. The operative temperature and the level of exercise intensity have an intuitive effect on thermal perception.Taking the moment at the end of the exercise as an example, the TSV increased by about 0.6 (S.D. = 0.258) with a 5℃ increase in the temperature, and the TSV increased by about 0.9 (S.D. = 0.307) with a 1.5 km/h increase in walking speed.
2. The local skin temperature decreased and then increased during the exercise, and gradually decreased after the further increase during post-exercise. The Tskin of calf was affected by the intensity of interval exercise and temperature, and showing a high correlation with TSV (*r* = 0.847).
3. The dynamic characteristics of HR and TSV showed synchrony in all temperature sets. The thermal environment’s impact on HR was not significance. Temperature and exercise intensity had an effect on HRV. HRV was higher during post-exercise than when exercising, and increasing temperature and activity intensity resulted in a higher HRV while exercising.
4. A significant positive correlation was observed between SFI and TSV, whereas the accuracy of estimating TSV by SFI was low when SFI was not greater than 0.5. SFI was not applicable to predicting TSV for hypothermia or low-intensity exercise.
5. Based on the dynamic characterization of physiological indicators and their effects on TSV, combined with the convenience, immediacy, and accuracy of parameter acquisition and evaluation, the HR and skin temperature of calf were selected as prediction parameters of thermal sensation during exercise. The corresponding prediction equations were constructed, which were found to account for 89.8% of changes in the thermal sensation.

## Supplementary Information

Below is the link to the electronic supplementary material.

Supplementary file1 (DCOX 176 KB)

## Data Availability

The data presented in this study are available on request from the corresponding author.
