# Supplementary material for: Experimental study on physiological responses during interval exercise and the effects on thermal perception
Source: Int J Biometeorol. 2025 Jul 24;69(10):2767–79. doi: 10.1007/s00484-025-02993-6 (PMC12540544; doi:10.1007/s00484-025-02993-6)
Supplement: Supplementary file 1 — Supplementary file1 (DCOX 176 KB) [file 484_2025_2993_MOESM1_ESM.docx]

**Appendix 1 Abbreviations**

TSV Thermal sensation vote

SFI Sweat feeling index

HSR Heat storage rate, W/m^2^

HR Heart rate, bpm

HRV Heart rate variation

T_op_ Operative temperature, ℃

T_skin_ Skin temperature, ℃

mT_skin_ Mean skin temperature, ℃

T_chest_ Skin temperature of chest, ℃

T_back_ Skin temperature of back, ℃

T_upperarm_ Skin temperature of arm, ℃

T_calf_ Skin temperature of calf, ℃

BMI Body mass index, kg/m^2^

BMR Basal metabolic rate, kcal/day

**Appendix 2 Supplementary figures and tables.**

**Fig. S1** Steady HRV for interval exercise in different thermal environment.

**Table S1** Environmental parameters in the controllable thermal chamber.

|  | T_op_ (℃) | Measured air  temperature (℃) | Measured wind speed (m/s) | Measured relative humidity (%) | Illumination (Lux) |
| --- | --- | --- | --- | --- | --- |
| Group 1 | -5 | -5.4 ±0.3 | 0.5 ±0.2 | 62 ±3.4 | 300 |
| Group 2 | 5 | 4.9 ±0.2 | 0.5 ±0.1 | 58 ±5.3 | 300 |
| Group 3 | 15 | 15.3 ±0.1 | 0.5 ±0.2 | 48 ±3.6 | 300 |
| Group 4 | 25 | 27.4 ±0.3 | 0.5 ±0.2 | 51 ±3.1 | 300 |
| Group 5 | 35 | 34.8 ±0.1 | 0.5 ±0.1 | 58 ±4.7 | 300 |

**Table S2** Correlation analysis between physiological indicators and TSV

|  | **Local skin temperature** | | | | | | **Temperature** | **HR** | **SFI** | **HRV** | **HSR** |
| --- | --- | --- | --- | --- | --- | --- | --- | --- | --- | --- | --- |
|  | Chest | Back | Upperarm | Calf | Forehead | mean |  |  |  |  |  |
| r | 0.676 | 0.803 | 0.720 | 0.847 | 0.817 | 0.815 | 0.821 | 0.526 | 0.883 | 0.413 | 0.829 |
| Sig. | 0.000 | 0.000 | 0.000 | 0.000 | 0.000 | 0.000 | 0.000 | 0.000 | 0.000 | 0.000 | 0.000 |

**Table S3** Coefficients of equation 1

| Model | Unstandardized Coefficients | | Standardized Coefficients | t | Sig. | Correlations | | | Collinearity Statistics | |
| --- | --- | --- | --- | --- | --- | --- | --- | --- | --- | --- |
|  | B | Std. Error | Beta |  |  | Zero-order | Partial | Part | Tolerance | VIF |
| (Constant) | -11.786 | 0.372 |  | -31.683 | 0.000 |  |  |  |  |  |
| Tskin of calf | 0.297 | 0.011 | 0.764 | 26.231 | 0.000 | 0.838 | 0.922 | 0.753 | 0.973 | 1.028 |
| HR | 0.030 | 0.002 | 0.450 | 15.450 | 0.000 | 0.576 | 0.813 | 0.444 | 0.973 | 1.028 |
| a. Dependent Variable: TSV | | | | | | | | | | |
